# Enhancing attention and emotion regulation in children with ADHD through AR digital picture books: a structural equation modeling approach

**DOI:** 10.3389/fpsyg.2025.1613728

**Published:** 2026-01-06

**Authors:** Rongming Yang, Weibo Sun, Xinwei Liu, Yuanfang Cao

**Affiliations:** 1 Graduate School of Global Convergence, Kangwon National University, Chuncheon, Republic of Korea; 2 Suzhou University of Technology, Suzhou, China

**Keywords:** augmented reality, ADHD, emotion regulation, attention enhancement, learning motivation, structural equation modeling

## Abstract

This study investigates the effectiveness of augmented reality (AR) digital picture books in improving attention and emotional regulation among children with Attention Deficit Hyperactivity Disorder (ADHD). Grounded in self-determination theory and emotion regulation frameworks, a five-variable structural model was constructed: AR motivation → learning motivation → attentional focus → emotional regulation → behavioral improvement. Cognitive load and its interaction effects were introduced as moderating variables. Forty children aged 9–11, including 20 diagnosed with ADHD and 20 typically developing children, participated in a 2-week AR picture book intervention. Quantitative data were collected via student-, teacher-, and parent-rated instruments. *T*-tests, MANOVA, partial least squares structural equation modeling (PLS-SEM), and multigroup analysis (PLS-MGA) were used for statistical analysis. The results indicated that AR picture books showed potential to improve learning motivation, attentional focus, and emotional regulation among ADHD children, ultimately enhancing behavioral outcomes. Learning motivation and attention served as significant mediators between AR experiences and emotion regulation. Cognitive load exhibited a negative moderating effect on the attentional-emotional pathway, with divergent patterns observed between ADHD and non-ADHD groups. These findings provide empirical support for the application of AR-based multisensory interaction design in special education interventions and offer practical insights into personalized cognitive-adaptive instructional design for inclusive learning environments.

## 1 Introduction

Attention Deficit Hyperactivity Disorder (ADHD) is among the most prevalent neurodevelopmental conditions in children, characterized by persistent patterns of inattention, hyperactivity, and impulsivity (Thapar et al., 2012). These symptoms often impair children’s ability to sustain focus and regulate emotions, leading to substantial difficulties in academic environments (Harpin, 2005; Zima et al., 2000). Epidemiological studies estimate that approximately 8.4% of children globally are diagnosed with ADHD, underscoring its widespread and long-term impact on school-age populations (Danielson et al., 2018; Simon et al., 2009).

Reading is a foundational skill for knowledge acquisition and social participation, closely linked to children’s cognitive development and psychological wellbeing (Castles et al., 2018). Early deficits in reading ability can pose serious risks to later academic success and cognitive growth (Hulme and Snowling, 2013). According to the Progress in International Reading Literacy Study (PIRLS), attentional capacity is a key determinant of both reading performance and frequency (Marôco, 2021). While reading competence is positively associated with reading motivation, recent national reports indicate that Chinese adolescents continue to exhibit low levels of reading interest and are limited by insufficient content diversity (China Education Online, 2024). Notably, reading difficulties and attentional impairments often co-occur and display overlapping cognitive and behavioral characteristics with ADHD (Mayes et al., 2000).

Traditional classroom instruction often depends heavily on sustained attention and linear information delivery, which can exacerbate learning barriers for children with attentional challenges. Similarly, conventional picture books may fail to capture attention due to their limited visual engagement and fragmented narrative structure. In recent years, the advent of digital technologies has opened new opportunities for neurodiverse learners by fostering increased engagement and deeper cognitive involvement (Chen et al., 2022). Among these innovations, augmented reality (AR)—renowned for its interactive, multisensory, and immersive features—has gained significant traction within the field of special education (Radu, 2014).

Augmented reality (AR) digital picture books, combining dynamic visuals, auditory narration, and touch-based interaction through mobile devices, offer a novel multisensory approach to children’s learning. Compared to traditional picture books, AR-based materials significantly enhance learners’ motivation, attention, and immersion (Yeh et al., 2022), while also improving reading comprehension and expressive language skills (Şimşek and Direkçi, 2023).

Despite these benefits, the application of AR tools for children with Attention Deficit Hyperactivity Disorder (ADHD) remains understudied, with few empirical studies offering in-depth analysis (Chen et al., 2022; Sanku et al., 2023). Most current interventions for ADHD still rely primarily on behavioral therapy or pharmacological treatment, leaving a gap in the literature regarding how educational technologies—particularly AR—can support attention control and emotional regulation in neurodiverse learners.

Emerging research highlights the promise of gamified and immersive AR tools in promoting self-regulation and learning outcomes among children with ADHD. Doulou and Drigas (2022) reported that AR and VR environments facilitate metacognitive development and emotional self-awareness, key domains in executive functioning. Similarly, Tosto et al. (2021) found that AR literacy programs not only improved reading proficiency but also enhanced emotional engagement and motivation in children diagnosed with ADHD. Goharinejad et al. (2022) further noted that while immersive XR technologies support attentional regulation, their effectiveness depends on factors such as cognitive load and sensory sensitivity.

Although existing studies affirm the educational benefits of AR in general populations (Radu, 2014; Yeh et al., 2022), there is a notable lack of systematic investigation into how AR picture books can meet the unique cognitive and emotional needs of children with ADHD (Chen et al., 2022). Previous research has largely focused on typically developing children, with limited attention given to ADHD-specific pathways, model validation, or multi-dimensional behavioral impacts (Psyrra et al., 2023). Moreover, the mediating and sequential mechanisms—such as the roles of learning motivation, attention focus, and emotion regulation—have not been sufficiently addressed in prior empirical frameworks.

To address these gaps, the present study constructs and validates a structural equation model (SEM) that examines how AR picture books influence behavioral improvement via sequential effects on motivation, attention, and emotion regulation. Grounded in Self-Determination Theory (Ryan and Deci, 2000, 2017), which posits motivation as a key driver of regulation and wellbeing, this study explores these mechanistic pathways. Using a task-based AR picture book system and a sample of 40 children (ADHD and typically developing), this study provides empirical evidence for the differential impact of AR-based interventions and proposes a scalable digital framework tailored to inclusive education environments.

## 2 Literature review

### 2.1 Augmented reality picture books and learning motivation

Augmented Reality (AR) refers to a technology that superimposes digital content onto the physical world, thereby enriching sensory engagement through visual, auditory, and tactile channels (Azuma, 1997). Owing to its immersive and interactive nature, AR has garnered considerable attention in education, where it has been shown to enhance learner engagement, support individualized learning, and improve memory retention (Radu, 2014). Empirical research indicates that AR environments significantly boost learners’ motivation, cognitive engagement, and comprehension by synchronizing multisensory inputs into a cohesive learning experience (Akçayır and Akçayır, 2017; Radu, 2014). Within the spectrum of AR-enhanced educational tools, AR digital picture books—which combine traditional narratives with 3D animations, audio narration, and interactive elements—have demonstrated considerable promise, particularly in early childhood and special education contexts (Cheng and Tsai, 2014; Tosto et al., 2021).

Since 2012, the global publication of AR books has expanded steadily, with many scholars viewing AR integration as a key trajectory in the evolution of digital publishing (Gudinavičius and Markelevičiūtė, 2020). These picture books are characterized by immersive storytelling that blends interactive animations, narrated explanations, and three-dimensional graphics to enhance learner engagement (Yeh et al., 2022).

For example, Cheng and Tsai (2014) developed an AR book entitled *YuYu’s Adventure*, which allowed children to explore virtual models from diverse angles, thereby encouraging parent–child interaction and enhancing cognitive development. In a multicultural setting, Chen et al. (2022) observed that AR storybooks incorporating ecological role-play scenarios significantly improved children’s pro-environmental attitudes and behaviors. Psyrra et al. (2023) developed an AR-driven literacy platform tailored for children with ADHD. Their results revealed notable improvements in reading behavior, attentional regulation, and task perseverance, facilitated by real-time word recognition and guided interaction.

Wu et al. (2013) reported that gamified AR learning environments increase intrinsic motivation while mitigating classroom distractions. Cheng and Tsai (2014) further emphasized that AR-supported reading scenarios foster both emotional engagement and cognitive advancement. Yilmaz et al. (2021), through a quasi-experimental design, demonstrated that AR storybooks substantially enhance children’s attitudes toward reading and their storytelling fluency. A growing body of research (e.g., Tzima et al., 2019; Kucuk et al., 2016; Yoon and Wang, 2020) corroborates the effectiveness of AR books in enhancing vocabulary development, reading engagement, and comprehension across varying age ranges and learner demographics.

### 2.2 Multisensory interaction and attentional control

Multisensory interaction designs—integrating visual, auditory, and tactile feedback—have been shown to significantly enhance attentional engagement in educational contexts (Billinghurst et al., 2015; Sankaranarayanan et al., 2022). In AR-based learning environments, the parallel processing of sensory input reduces cognitive load and supports sustained attention (Dunleavy and Dede, 2014; McMahon et al., 2016). Interactive AR content, especially when embedded with task-oriented elements such as manipulating 3D models or solving narrative-based challenges, has demonstrated efficacy in improving reading comprehension, attentional control, and executive functioning—particularly for children with ADHD (Yang et al., 2025; Zhou and Yadav, 2017; Hautala et al., 2022; Park et al., 2023).

From a psychological perspective, learning motivation and attentional focus serve as critical mediators of emotion regulation and behavioral development (Bandura, 1997; Mayer, 2005). When children experience a sense of self-efficacy and perceived control, their emotional resilience and frustration tolerance improve significantly (Durlak et al., 2011). Within AR-enhanced learning contexts, increased motivation and focus can act as psychological buffers, helping learners manage cognitively demanding tasks more effectively. Ronimus et al. (2022) further noted that children exhibit greater emotional stability and social adaptability when learning tasks are perceived as meaningful and achievable. Thus, by activating both motivational and attentional mechanisms, AR technology offers indirect but promising pathways for supporting emotion regulation in children with ADHD.

### 2.3 Emotion regulation challenges and intervention strategies for children with ADHD

Children with Attention Deficit Hyperactivity Disorder (ADHD) commonly experience significant difficulties in emotion regulation, including heightened reactivity, impulsivity, and delayed emotional recovery (Barkley, 2015; American Psychiatric Association, 2013). Such emotional dysregulation often leads to disruptive classroom behaviors and challenges in social adaptation, while traditional educational settings frequently fail to meet their emotional and attentional needs. According to Bandura (1977) self-efficacy theory, a learner’s belief in their own ability to complete academic tasks promotes emotional stability and task persistence. Within AR-supported learning environments, interactive and immersive elements can activate intrinsic motivation, thereby enhancing attentional control and emotional regulation (Rueda et al., 2005; Mayer, 2005).

Recent studies have increasingly focused on the role of immersive technologies in emotion-centered interventions. Tosto et al. (2021) and Sulaiman and Shaid (2023) demonstrated that AR environments featuring real-time feedback and adaptive sensory modulation can reduce emotional volatility among children with ADHD. Similarly, Obradović et al. (2012) highlighted that self-monitoring and feedback-based systems foster emotional stability and behavioral regulation. Ronimus et al. (2022) found that when children perceive learning tasks as meaningful and achievable, they display more stable emotional responses and stronger social adaptability. Durlak et al. (2011) also emphasized that perceived control and self-efficacy enhance emotional resilience and frustration tolerance. Within AR-mediated tasks, heightened motivation and attentional focus may serve as psychological buffers, helping children manage emotionally demanding scenarios more effectively.

However, cognitive load theory warns that overly complex sensory input or poorly structured feedback can diminish motivation and even cause cognitive fatigue (Sweller et al., 2011; Zheng, 2022). Consequently, the design of AR picture books should balance interactivity with simplicity to optimize learning and emotional outcomes. Prior research suggests that digital reading environments can increase children’s engagement and learning interest (Piotrowski and Krcmar, 2017; Ghalebandi and Noorhidawati, 2019), while Boyask et al. (2023) further noted that playful and interactive learning experiences extend attention spans and promote emotional regulation. Despite the broad application of AR in general education, its systematic use for addressing ADHD-related emotional and attentional challenges remains underexplored. Notably, there is still a lack of integrative frameworks capable of modeling mediating and moderating mechanisms. To address this research gap, the present study proposes a structural equation model (SEM) that links AR picture book engagement to behavioral improvement through learning motivation, attentional control, and emotional regulation, incorporating perceived cognitive load as a key moderating variable. This framework aims to provide empirical support for the educational and psychological value of AR interventions for children with ADHD and to enrich theoretical discourse in this emerging field.

## 3 Theoretical framework and hypotheses

### 3.1 Conceptual model and research hypotheses

This study proposes a theoretically grounded framework to examine how augmented reality (AR) picture books impact behavioral outcomes in children diagnosed with Attention Deficit Hyperactivity Disorder (ADHD). Integrating concepts from self-determination theory, task-based learning, cognitive load theory, and emotion regulation models, we construct a multi-pathway conceptual model. The model posits that AR engagement enhances both learning motivation and attentional focus, which in turn foster improved emotion regulation and subsequent behavioral enhancement. Additionally, perceived cognitive load is introduced as a moderating variable, and two indirect pathways—via learning motivation and attentional focus—are hypothesized and empirically tested.

### 3.2 Proposed hypotheses

#### 3.2.1 Main effect hypotheses

*H1:* The use of AR picture books will positively influence learning motivation among children with ADHD.

*H2:* The use of AR picture books will positively influence attentional focus among children with ADHD.

*H3:* Learning motivation will positively influence emotion regulation among children with ADHD.

*H4:* Attentional focus will positively influence emotion regulation among children with ADHD.

*H5:* Emotion regulation will positively influence behavioral improvement among children with ADHD.

#### 3.2.2 Moderation hypothesis

*H6:* Perceived cognitive load moderates the relationship between learning motivation and emotion regulation. When cognitive load is low, the positive impact of motivation on emotion regulation will be stronger.

#### 3.2.3 Mediation hypotheses

*H7a:* AR picture books will indirectly influence emotion regulation and behavioral outcomes by increasing learning motivation.

*H7b:* AR picture books will indirectly influence emotion regulation and behavioral outcomes by improving attentional focus.

The study involved 40 fifth-grade students aged 9–11, drawn from two distinct elementary school settings. The experimental group comprised 20 children from a special education school in Mengcheng County, Anhui Province, all identified by certified educators as exhibiting core characteristics of ADHD. The control group consisted of 20 typically developing peers from a public school in Hangzhou, Zhejiang Province, matched in age and academic background. The 9–11 age range was chosen as it represents a pivotal developmental stage for attention regulation, reading comprehension, and emotion management—skills particularly responsive to visually enriched, task-based interventions such as AR picture books.

### 3.3 Educational materials: AR picture book design

The development of the AR picture book was guided by the classification framework of the Progress in International Reading Literacy Study (PIRLS), which considers reading purposes, age-appropriate preferences, and content relevance. The final version adopted a visually dominant format to accommodate both typically developing children and those with ADHD, enabling observation of attentional and emotional regulation responses through visualized, task-oriented stimuli.

The design process involved several iterative team discussions. All team members had academic training in art education and hands-on experience in early childhood instruction, which facilitated consensus on the book’s visual language, including its color palette, character design, and narrative pacing. Three alternative design prototypes were initially proposed:

#### 3.3.1 Design 1: interactive, story-driven, task-based AR book

This primary design was based on task-based learning (TBL) theory and leveraged AR’s interactive capabilities. It combined a narrative structure with multisensory feedback to create a tiered intervention experience. The storyline centers on a lion cub named “Little Brave Lion” who embarks on a quest to “find the stars.” Along this journey, the character completes a series of structured missions—such as helping friends retrieve lost items, navigating “emotional clouds,” and unlocking symbolic “courage masks.” These tasks were designed to foster learning motivation, sustained attention, and emotional self-regulation.

#### 3.3.2 Design 2: emotion-focused AR book based on sensory guidance

Grounded in Social and Emotional Learning (SEL) principles, this design emphasized emotion recognition and expression as foundational elements. It utilized multimodal sensory cues and a stress-free, intuitive interface to create a psychologically safe space where children with ADHD could explore and regulate their emotional states.

#### 3.3.3 Design 3: attention training maze-based AR book

This design drew upon attention control theory and featured persistence-based challenge design. Through a sequential maze interface, children were prompted to sustain visual focus and solve increasingly complex problems. The core emphasis was on building attentional stamina, with emotion regulation elements incorporated as a secondary objective.

**FIGURE 1:**
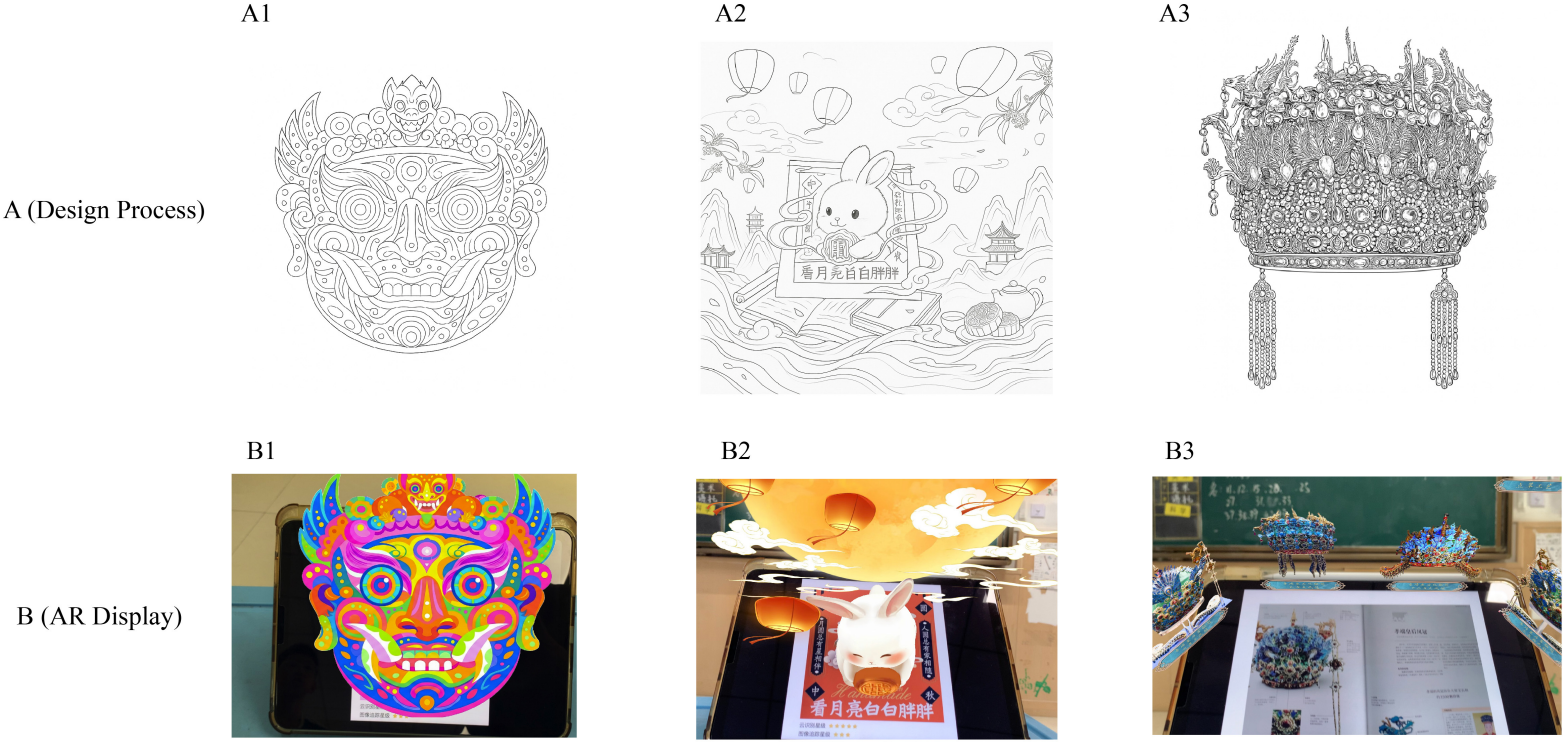
Design evolution and augmented reality (AR) implementation of the digital picture book assets. **(A)** (Design Process): The original line art and structural sketches for (A1) the Mythical Beast Mask, (A2) “The Call of the Rabbit” illustration, and (A3) the Phoenix Crown. (B) (AR Display): The corresponding final AR effects displayed on a tablet, showing (B1) the interactive mask, (B2) the animated rabbit scene, and (B3) the 3D crown model overlaid on the physical book.

Design 1 was ultimately selected for the intervention. The story-driven AR book was divided into modular chapters, each containing a “lightweight task” with clearly defined objectives and minimal complexity. This structure was tailored to the cognitive profiles of children with ADHD, particularly their short attention spans and developing executive functions. The design followed a pedagogical sequence of “hands-on → reflection → expression,” supported by immersive AR feedback to cultivate intrinsic engagement (Figure 1).

Moreover, emotion-related elements were embedded directly into the storyline through observation prompts, affective choices, and real-time feedback mechanisms. This integration allowed the intervention to simultaneously target learning motivation, attentional control, and emotional regulation, facilitating both operational implementation and empirical measurement of the behavioral constructs.

### 3.4 System development and deployment

This study developed a three-chapter interactive augmented reality (AR) picture book system based on image recognition technology. The system was implemented by integrating the Unity3D game engine with the Vuforia Software Development Kit (SDK). Designed as a narrative-centered educational intervention, the AR picture book blends immersive storytelling with multimodal interaction, allowing for precise synchronization between virtual content and tangible learning materials.

The system development process followed a three-stage structure:

*(1) Text Design and Structural Planning* During the initial planning phase, the research team outlined the educational objectives and intervention logic of the picture book. The structure was segmented into three core chapters, each incorporating task checkpoints and interactive components aligned with specific pedagogical goals. This ensured a clear cognitive progression and instructional coherence throughout the experience.

*(2) Visual Asset Creation and Feature Integration* In the asset development phase, the team employed Midjourney (for AI-generated visual content) and ChatGPT (for narrative ideation) to produce foundational illustrations and storyline prompts. These visuals were subsequently refined using Adobe Photoshop and Illustrator to ensure stylistic consistency and layout precision. To support immersive storytelling, 3D assets—including models, animations, and props—were created using Blender and Maya. Supplementary multimedia assets, such as voice-acted character lines, narrative instructions, and auditory feedback cues, were also developed to enhance user engagement and system usability.

*(3) System Implementation and Platform Deployment* The final interactive system was developed using Unity3D and the Vuforia SDK, incorporating key functions such as image recognition, 3D object rendering, animation triggering, and synchronized audio output. The system was deployed through Kivicube—a prominent domestic WebAR platform—enabling cross-device compatibility with smartphones, tablets, and smart classroom terminals. Using Kivicube’s online scripting tools, features such as button activation, drag-and-drop functionality, and real-time feedback mechanisms were integrated. To accommodate the cognitive needs of children with ADHD, the system was optimized in terms of feedback latency, task pacing, and user interface simplicity. Special attention was given to interface stability, visual accessibility, and operational clarity to ensure smooth deployment in educational settings.

### 3.5 Participants and grouping

A total of 40 fifth-grade students aged 9–11 were recruited from two distinct elementary school settings. The experimental group comprised 20 children from a special education school in Mengcheng County, Anhui Province, all of whom were identified by certified educators as exhibiting behavioral characteristics consistent with Attention Deficit Hyperactivity Disorder (ADHD). The control group consisted of 20 typically developing children enrolled in a public elementary school in Hangzhou, Zhejiang Province. Due to the distinct school settings, a non-randomized matched-pair design was employed. Participants in the control group were carefully matched with the experimental group based on age and academic background to ensure baseline comparability.

The selected age range of 9–11 years reflects a critical developmental window for cultivating attention control, reading comprehension, and emotion regulation—core competencies that are especially responsive to visually enriched and task-oriented interventions. To ensure procedural equity and maintain consistency in the delivery of the AR intervention, several strict control measures were implemented. First, both groups utilized the same types of digital devices—including tablets, smartphones, and AR-enabled classroom displays—during all experimental tasks. Second, all supervising educators underwent a standardized training session prior to the study to ensure uniform instruction and guidance across both locations. Third, a procedural checklist was used to monitor adherence to the intervention protocol.

Before the intervention, all participants were assessed for their ability to operate the AR picture book system and were confirmed to have the requisite technical competencies and a positive disposition toward participation. Informed consent was obtained from the legal guardians of all participants in accordance with ethical research protocols. To enhance the study’s social inclusion dimension, a “peer-supported learning environment” was established. This framework enabled children from both mainstream and special education backgrounds to engage in shared content exploration and synchronous interactive learning across institutional boundaries.

## 4 Measures and experimental procedure

### 4.1 Variables and instruments

This study adopted a structured measurement framework comprising independent variables, mediating variables, moderating variables, and dependent outcomes. All instruments were adapted from internationally validated scales, with minor modifications to ensure cultural and linguistic suitability for the target population. A five-point Likert scale (1 = strongly disagree to 5 = strongly agree) was applied to all items, and all instruments exhibited strong internal consistency (Cronbach’s alpha > 0.80).

#### 4.1.1 Independent variables

The core independent variable was AR engagement, defined as the degree of student involvement and intrinsic motivation during interaction with AR picture books. This construct was measured using items adapted from the Self-Determination Theory (SDT) framework proposed by Champ et al. (2023), with a particular focus on task relevance and autonomous learning drive. In addition, perceived cognitive load was conceptualized both as a contextual independent variable and a moderating factor. It was assessed using a simplified version of the NASA Task Load Index (NASA-TLX) adapted by Mitsea et al. (2023), capturing the psychological effort experienced during AR-based learning tasks.

#### 4.1.2 Mediating variables

Two core mediating variables were included: learning motivation and attentional focus. Learning motivation was evaluated using the multidimensional scale developed by He et al. (2021), which assesses students’ cognitive engagement, emotional investment, and behavioral participation in academic tasks. Attentional focus was measured through items drawn from the attentional regulation framework validated by Zheng et al. (2021), reflecting the ability to sustain concentration and resist distraction during reading activities.

#### 4.1.3 Dependent variables

Two outcome variables were assessed. First, emotion regulation was measured using an adapted version of the Cognitive Emotion Regulation Questionnaire (CERQ) by Zhao and Zhang (2024), which evaluates students’ ability to manage emotional responses in academic contexts. Second, behavioral improvement—referring to observable changes in classroom conduct and self-regulation—was measured through teacher-rated items adapted from the behavioral evaluation framework developed by Keshav et al. (2019).

### 4.2 Experimental procedure

This study adopted a pretest–posttest quasi-experimental design involving two cohorts: children diagnosed with Attention Deficit Hyperactivity Disorder (ADHD) and typically developing children (TDC). Each group comprised 20 students aged 9–12, recruited respectively from a public elementary school and a specialized education institution.

The primary objective was to assess whether AR-enhanced digital picture books could significantly improve attentional focus and emotion regulation in children with ADHD, relative to their typically developing counterparts. Both groups were evaluated using identical validated instruments before and after a structured AR picture book interaction session. Each test session was supervised by two trained educators: one facilitated task guidance, while the other observed student behavior and recorded ratings using standardized assessment tools.

**FIGURE 2:**
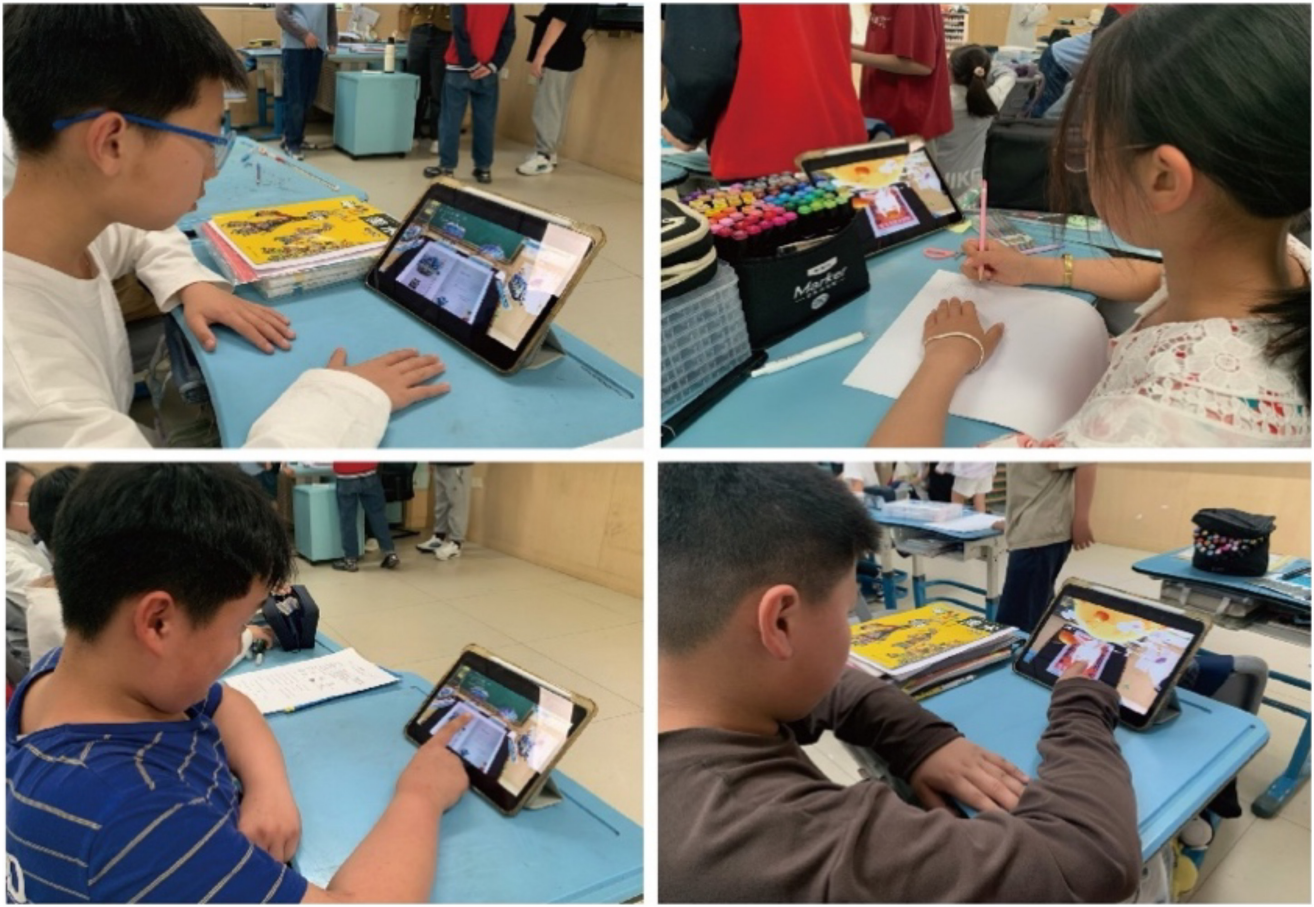
Testing environment schematic.

**FIGURE 3:**
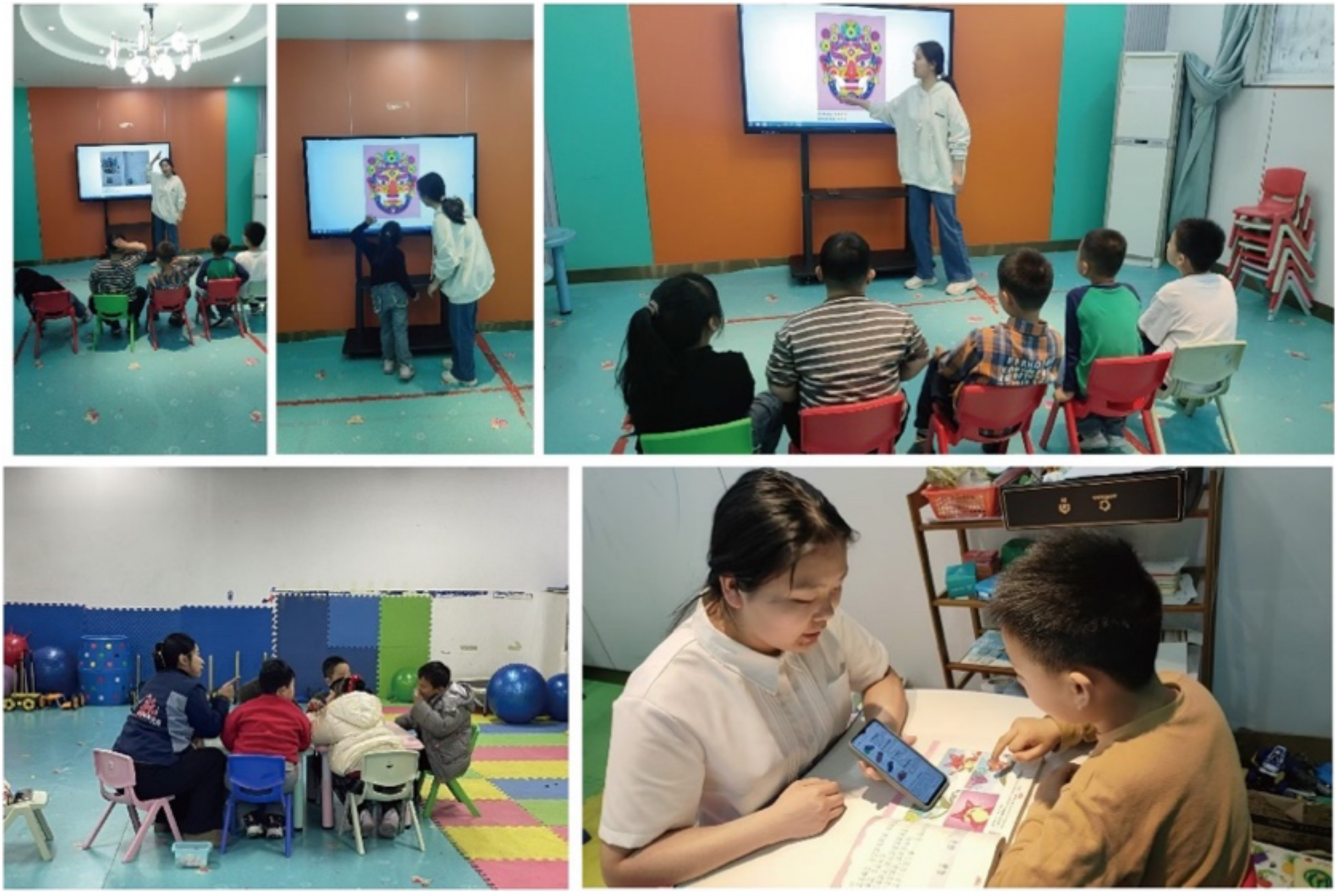
Classroom photo caption.

A visual depiction of the testing environment is provided in Figures 2, 3.

Prior research highlights teachers as reliable external evaluators of children’s self-regulation, attention, and social behavior within school environments. Teacher ratings have shown strong predictive validity for emotional disturbances, aggression, and behavior-related developmental outcomes (Teglasi et al., 2015; Whipp et al., 2021). Accordingly, teacher-based evaluations in this study provide strong external validity for assessing behavioral and emotional outcomes in both ADHD and typically developing students under ecologically valid classroom conditions.

## 5 Results

### 5.1 Paired sample *t*-tests

To assess the effectiveness of the AR picture book intervention, paired sample *t*-tests were conducted separately for each group. The analysis compared pretest and posttest scores across six core psychological constructs: AR engagement motivation, learning motivation, attentional focus, emotion regulation, behavioral improvement, and perceived cognitive load.

#### 5.1.1 Results for the typically developing children group

Among the typically developing children (TDC), significant improvements were observed across all measured variables from pretest to posttest. In total, 19 items spanning six subscales were analyzed. As presented in Table 1, all four items measuring AR engagement motivation showed statistically significant increases after the intervention (*t* = 2.775–6.990, *p* < 0.05), indicating that AR-based interaction effectively enhanced learner motivation. Similarly, all three items assessing learning motivation demonstrated significant gains (*p* < 0.05), suggesting that the AR experience successfully stimulated intrinsic academic interest. In terms of attentional focus, a clear increase in posttest scores was recorded (*t* = –2.896 to –6.484, all *p* < 0.01), implying that the intervention supported improvements in sustained attention.

**TABLE 1:** Paired sample *t*-test results for the typically developing children (TDC) group.

Variables	Mean	Standard deviation	Standard error mean	*t*-value	*p*-value
AR1 - AR1	−1.200	0.768	0.172	−6.990	0.000
AR2 - AR2	−1.050	0.826	0.185	−5.688	0.000
AR3 - AR3	−1.100	0.912	0.204	−5.395	0.000
AR4 - AR4	−0.750	1.209	0.270	−2.775	0.012
LM1 - LM1	−0.550	0.887	0.198	−2.773	0.012
LM2 - LM2	−1.700	0.979	0.219	−7.768	0.000
LM3 - LM3	−0.750	1.118	0.250	−3.000	0.007
AF1 - AF1	−1.450	0.686	0.153	−9.448	0.000
AF2 - AF2	−1.250	0.910	0.204	−6.140	0.000
AF3 - AF3	−0.700	1.081	0.242	−2.896	0.009
ER1 - ER1	−1.200	0.894	0.200	−6.000	0.000
ER2 - ER2	−1.650	1.040	0.233	−7.095	0.000
ER3 - ER3	−1.450	0.686	0.153	−9.448	0.000
BO1 - BO1	−0.900	0.968	0.216	−4.158	0.001
BO2 - BO2	−0.700	1.031	0.231	−3.036	0.007
BO3 - BO3	−1.150	0.988	0.221	−5.205	0.000
CL1 - CL1	−1.100	0.912	0.204	−5.395	0.000
CL2 - CL2	−1.400	0.940	0.210	−6.658	0.000
CL3 - CL3	−0.650	0.745	0.167	−3.901	0.001

The most substantial change was observed in the emotion regulation domain, with all items showing highly significant improvement (*p* < 0.001), reflecting the AR intervention’s emphasis on emotional engagement and interactive responsiveness.

Both behavioral improvement and perceived cognitive load showed favorable shifts following the intervention. Specifically, items measuring perceived cognitive load revealed a consistent decrease in posttest scores (negative *t*-values), indicating that participants experienced reduced cognitive strain during or after the AR experience. This reduction in perceived load supports the interpretation that AR-based tasks not only boost engagement and motivation but also mitigate subjective mental effort. As shown in Figure 3, the AR picture book intervention led to significant gains across multiple psychological domains—most notably in attentional focus, emotion regulation, and learning motivation. These findings establish a strong empirical foundation for subsequent structural equation modeling and exploration of underlying mechanisms.

#### 5.1.2 Results for the ADHD group

To evaluate the impact of the AR picture book intervention on children diagnosed with ADHD, paired sample *t*-tests were conducted to compare pre-intervention and post-intervention scores across six key psychological constructs: AR engagement motivation, learning motivation, attentional focus, emotion regulation, behavioral improvement, and perceived cognitive load.

As presented in Table 2, all items related to AR engagement motivation showed statistically significant improvements following the intervention (*t*-values ranging from -2.538 to -4.046, *p* < 0.05), indicating increased interest and engagement with the AR materials among children with ADHD.

**TABLE 2:** Paired samples test.

Variables	Mean	Standard deviation	Standard error mean	*t*-value	*p*-value
AR1 - AR1	−0.950	1.191	0.266	−3.567	0.002
AR2 - AR2	−0.950	1.050	0.235	−4.046	0.001
AR3 - AR3	−0.900	1.119	0.250	−3.596	0.002
AR4 - AR4	−0.900	1.586	0.355	−2.538	0.020
LM1 - LM1	−1.100	1.021	0.228	−4.819	0.000
LM2 - LM2	−1.300	1.261	0.282	−4.611	0.000
LM3 - LM3	−1.150	1.268	0.284	−4.056	0.001
AF1 - AF1	−1.300	1.302	0.291	−4.466	0.000
AF2 - AF2	−1.200	1.196	0.268	−4.485	0.000
AF3 - AF3	−0.700	1.129	0.252	−2.774	0.012
ER1 - ER1	−1.200	1.105	0.247	−4.857	0.000
ER2 - ER2	−1.700	1.380	0.309	−5.508	0.000
ER3 - ER3	−1.350	0.933	0.209	−6.469	0.000
BO1 - BO1	−1.250	1.164	0.260	−4.802	0.000
BO2 - BO2	−0.850	1.137	0.254	−3.344	0.003
BO3 - BO3	−0.900	1.165	0.261	−3.454	0.003
CL1 - CL1	−1.500	1.395	0.312	−4.807	0.000
CL2 - CL2	−1.150	1.309	0.293	−3.929	0.001
CL3 - CL3	−0.900	1.071	0.240	−3.758	0.001

Within the learning motivation subscale (Items B1–B3), all three items exhibited highly significant increases (*p* < 0.001), suggesting that the AR-based intervention effectively stimulated intrinsic motivation in ADHD participants. The attentional focus subscale also demonstrated a statistically significant improvement (*p* = 0.012), with an average increase of 1.20 points from pretest to posttest, supporting the efficacy of AR content in enhancing attentional resource allocation.

In the domain of emotion regulation, all items showed highly significant gains (*t* = –4.857 to –6.407, all *p* < 0.001), confirming the intervention’s practical effectiveness in improving emotional self-management and regulation skills. All items under the behavioral outcome dimension achieved statistical significance (*p* < 0.01), indicating positive changes in classroom behavior and task persistence among ADHD participants. Finally, a downward trend in perceived cognitive load scores was observed after the intervention, suggesting that children experienced reduced mental effort during AR-based tasks. This outcome aligns with the system’s design intent to provide cognitively accessible, low-pressure learning experiences for neurodiverse learners.

Collectively, the paired sample *t*-test results confirm that the AR picture book intervention produced broad and statistically significant improvements among children with ADHD. Notably, the intervention proved particularly effective in enhancing motivational engagement, attentional stability, emotional regulation, and perceived cognitive efficiency, highlighting its theoretical contribution and practical applicability.

### 5.2 Multivariate analysis of variance

To further assess overall group differences between typically developing children (TDC) and children with ADHD after the intervention, a Multivariate Analysis of Variance (MANOVA) was conducted. MANOVA was selected due to its ability to account for intercorrelations among dependent variables and to mitigate the risk of Type I error inflation associated with multiple univariate tests. This approach is well-suited for evaluating comprehensive differences across multiple outcome measures.

As presented in Table 3, all four multivariate test statistics—Wilks’ Lambda, Pillai’s Trace, Hotelling’s Trace, and Roy’s Largest Root—yielded statistically significant results, indicating a systematic and robust difference between the two groups across the six measured psychological dimensions.

**TABLE 3:** Results of MANOVA analysis.

Test methods	Value	*F*-value	*P*-value
Wilks’ Lambda	0.5787	4.0041	0.0041***
Pillai’s Trace	0.4213	4.0042	0.0039***
Hotelling Trace	0.7281	4.0046	0.0038***
Roy’s Root	0.7281	4.0041	0.0037***

***Indicates statistical significance at the *p* < 0.01 level.

Specifically, Wilks’ Lambda was 0.5787, with an associated *F*-value of 4.0041 and a significance level of *p* = 0.0041. A Wilks’ Lambda value below 0.60 typically reflects a moderate-to-strong multivariate effect, suggesting the presence of significant between-group differences in the overall outcome pattern. Consistent results were observed across other multivariate indices: Pillai’s Trace (*V* = 0.4213, *p* = 0.0039), Hotelling’s Trace (*T* = 0.7281, *p* = 0.0038), and Roy’s Largest Root (Θ = 0.7281, *p* = 0.0037), all of which reinforce the statistical robustness and validity of the model.

Taken together with the findings from the paired sample *t*-tests, the MANOVA offers a broader statistical perspective. Although the ADHD group demonstrated substantial improvements across most dimensions, their post-intervention profiles remained significantly different from those of the TDC group. These results support the hypothesis concerning group-level differences in intervention effectiveness (H4) and provide a solid foundation for subsequent multi-group analyses using structural equation modeling (PLS-MGA).

### 5.3 Multi-group analysis (PLS-MGA)

To further examine the structural relationships among latent variables within the proposed intervention model and to test the validity of the research hypotheses, this study employed Partial Least Squares Structural Equation Modeling (PLS-SEM) using SmartPLS 4.0. PLS-SEM is particularly well-suited for studies with relatively small sample sizes and complex model structures, making it ideal for exploratory and intervention-based research involving multivariate causal inference and prediction (Barroso et al., 2018).

Prior to model construction, data from the typically developing children (TDC) group and the ADHD group were merged into a single dataset. During the data preprocessing phase, missing values were examined and removed to ensure data completeness and integrity. A binary grouping variable was introduced to differentiate participants, with the TDC group coded as 0 and the ADHD group as 1. This enabled stratified model comparison within a unified analytic framework (Cheah et al., 2020).

**FIGURE 4:**
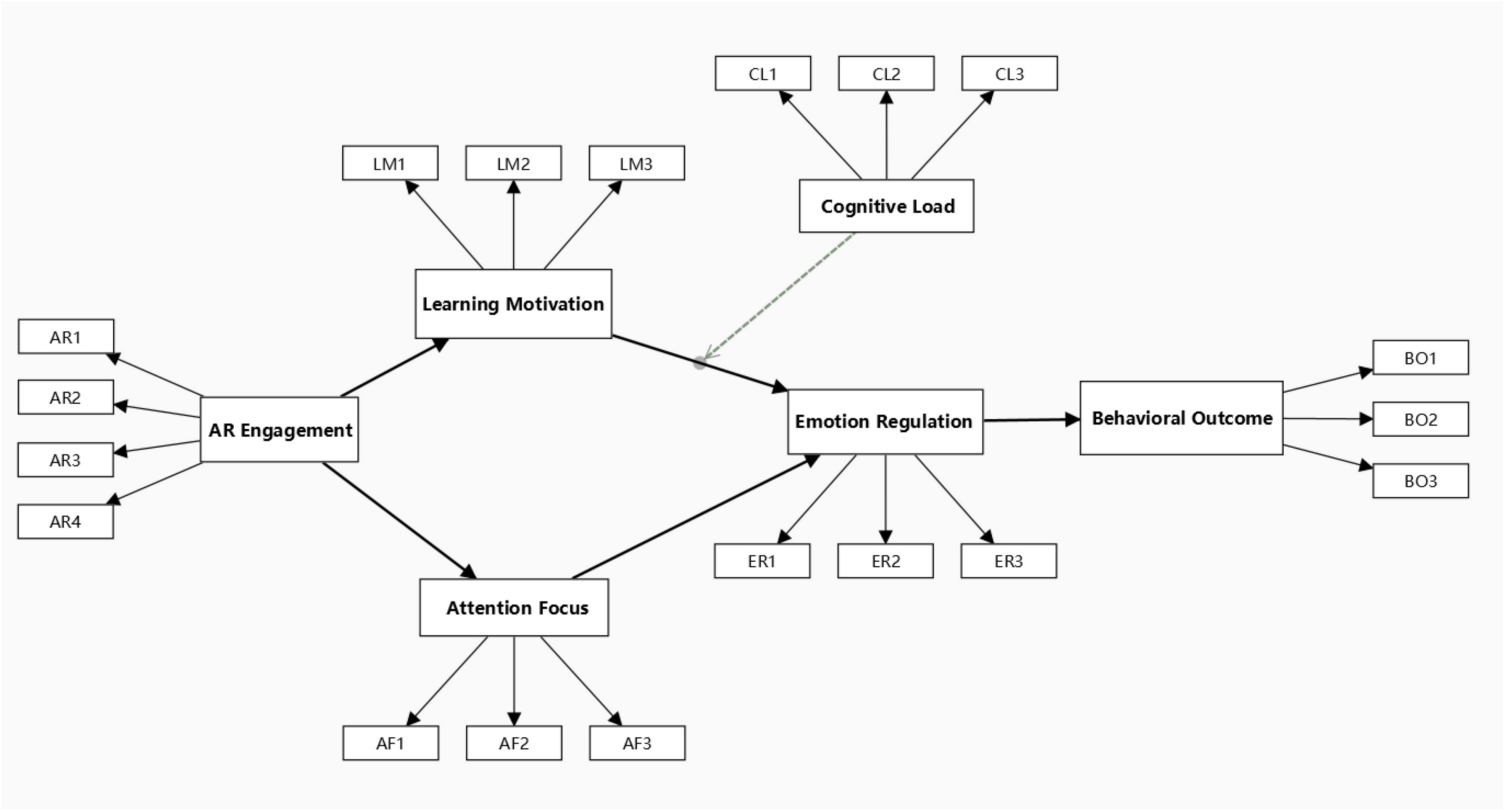
Constructing a hypothetical model of the effects of AR picture books on emotion regulation and behavioral cognition.

As emphasized by Cheah et al. (2020), PLS-MGA requires both subgroups to have equivalent model structures, including matched sample sizes, identical variable sets, and consistent item distributions. After validating these conditions, the structural model was constructed using the full dataset and executed in SmartPLS. The baseline model structure is presented in Figure 4. A preliminary test confirmed satisfactory model fit and convergence, indicating that the model met the criteria for multigroup path analysis and subsequent hypothesis testing.

Before proceeding with hypothesis testing, compositional invariance was assessed using the MICOM (Measurement Invariance of Composite Models) procedure. According to Cheah et al. (2020), non-significant *p*-values (*p* > 0.05) indicate that measurement invariance exists between groups. As presented in Table 4, all *p*-values exceeded the.05 threshold, confirming compositional invariance and validating the model’s suitability for cross-group comparisons.

**TABLE 4:** Results of measurement invariance assessment (MICOM).

Construct	Original correlation	Correlation mean	5.0% quantile	Permutation *p*-value
AR engagement	0.871	0.099	−0.711	0.969
Attention focus	0.662	0.285	−0.669	0.734
Behavioral outcome	0.921	0.177	−0.795	0.955
Cognitive load	−0.934	0.028	−0.947	0.057
Emotion regulation	0.963	0.034	−0.772	0.993
Learning motivation	−0.887	0.014	−0.831	0.055

Subsequently, permutation-based Multi-Group Analysis (PLS-MGA) was performed to determine whether structural path coefficients significantly differed between the two groups. This non-parametric permutation approach is among the five widely accepted techniques for group comparison, alongside Henseler’s bootstrapped MGA (Henseler et al., 2009), parametric testing (Keil et al., 2000), the Welch–Satterthwaite *t*-test (Welch, 1947), and permutation-based testing within the MICOM framework (Chin and Dibbern, 2010). In this study, the permutation-based MGA was chosen due to its robustness and high compatibility with the PLS-SEM analytical platform.

As presented in Table 5, the permutation-based analysis revealed significant differences in path coefficients between the ADHD and TDC groups across several key theoretical constructs. These findings reinforce the appropriateness of the multi-group intervention model and demonstrate that the AR picture book intervention exerts varying effects depending on group-specific characteristics. Accordingly, the results provide additional empirical support for Hypothesis H4, which posits differential intervention outcomes based on participant group.

**TABLE 5:** Analysis of variance (ANOVA) results.

Constructs	Configurational Invariance (Step 1)	Compositional Invariance (Step 2)		Partial Measurement Invariance	Original Differences	Equal Mean Assessment (Step 3a)		Original Differences	Equal Variance Assessment (Step 3b)		Full Measurement Invariance
AR engagement	YES	0.871	−0.711	YES	−0.619	−0.653	0.572	0.378	−0.91	0.954	YES/YES
Attention focus	YES	0.662	−0.669	YES	−0.553	−0.691	0.584	0.109	−0.773	0.769	YES/YES
Behavioral outcome	YES	0.921	−0.795	YES	−1.039	−0.67	0.609	0.087	−0.768	0.721	NO/YES
Cognitive load	YES	−0.934	−0.947	YES	−0.681	−0.636	0.655	0.112	−0.628	0.65	NO/YES
Emotion regulation	YES	0.963	−0.772	YES	−0.297	−0.638	0.624	0.492	−0.699	0.632	YES/YES
Learning motivation	YES	−0.887	−0.831	YES	−0.625	−0.638	0.599	0.58	−1.101	1.144	YES/YES

Step 1: Normally, this is automatically established. Step 2: The original correlation is higher than 5% and the permutation *p*-value is higher than 0.05. Step 3: (a) Not all confidence intervals of latent variable score means include the original differences value, so there is not equal means. (b) Not all confidence intervals of latent variable score variances include the original differences value, so there are not equal variances.

### 5.4 Structural path verification

To further examine the structural distinctions between the typically developing children (TDC) and the ADHD groups, this study utilized Partial Least Squares Multi-Group Analysis (PLS-MGA) to estimate and compare the path coefficients. Bootstrapping procedures were employed to evaluate the statistical significance of each path within the respective groups. Tables 6, 7 present the unstandardized path coefficients, bootstrapped standard errors (STDEVs), *t*-values, and corresponding *p*-values for all eight hypothesized relationships across the two cohorts.

**TABLE 6:** ADHD path test results.

Structural path	Path coefficient	Standard deviation (STDEV)	*t*-value	*p*-value	Result
AR→AF	−1.828	1.237	−3.437	0.000	YES/YES
AR→LM	−1.086	1.194	−3.750	0.000	YES/YES
AF→ER	0.712	1.247	−3.891	0.000	YES/YES
CL→ER	−0.547	1.284	−4.006	0.012	YES/YES
ER→BO	−0.358	1.213	−4.488	0.012	YES/YES
LM→ER	−0.387	1.257	−4.405	0.000	YES/YES
CL^∙^LM→ER	0.253	1.224	−3.945	0.007	YES/YES
CL^∙^AF→ER	−0.177	1.183	−4.146	0.000	YES/YES

**TABLE 7:** TDC path test results.

Structural path	Path coefficient	Standard deviation (STDEV)	*t*-value	*p*-value	Result
AR→AF	4.087	0.929	−5.212	0.002	YES/YES
AR→LM	1.839	0.959	−4.158	0.001	YES/YES
AF→ER	0.636	0.997	−4.678	0.002	YES/YES
CL→ER	0.240	1.048	−4.079	0.020	YES/YES
ER→BO	−0.992	0.918	−5.747	0.000	YES/YES
LM→ER	−0.600	0.923	−6.589	0.000	YES/YES
CL∙LM→ER	−0.077	0.949	−5.371	0.001	YES/YES
CL∙AF→ER	−0.019	0.893	−6.121	0.000	YES/YES

**FIGURE 5:**
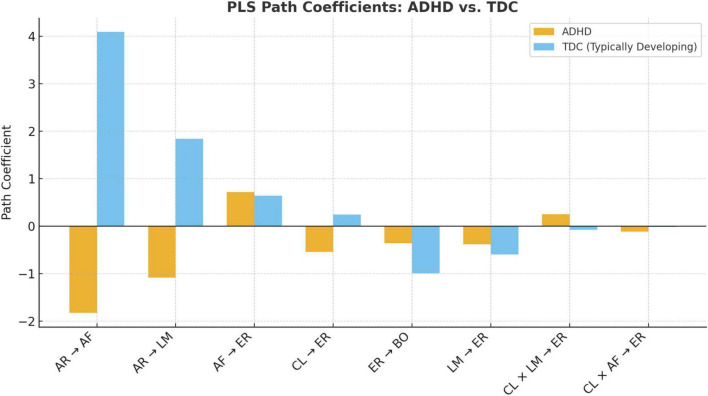
Comparison of path coefficients between ADHD and typically developing children (TDC) groups.

Additionally, Figure 5 offers a visual comparison of the path coefficients, clearly illustrating the variations in both direction and magnitude between the TDC and ADHD groups. This graphical representation facilitates the interpretation of group-specific dynamics and reinforces the importance of tailoring intervention strategies to the distinct cognitive and emotional profiles of each group.

The analysis revealed that most structural paths were statistically significant (*p* < 0.05) in both groups, offering robust empirical support for the proposed theoretical model. A summary of the core findings is presented below:

*H1*: AR Engagement → Attention Focus

A significant intergroup divergence was observed. For the ADHD group, the path coefficient was negative (β = –1.828, *p* = 0.000), whereas for the TDC group, it was strongly positive (β = 4.087, *p* = 0.002). This reversal indicates that children with ADHD may respond to AR stimuli in a contrasting manner compared to typically developing peers, thus providing partial support for H1.

*H2*: AR Engagement → Learning Motivation

Both groups exhibited significant positive associations (ADHD: β = –1.086; TDC: β = 1.839, both *p* < 0.01), validating the predictive role of AR engagement in enhancing learning motivation. H2 is supported.

*H3*: Attention Focus → Emotion Regulation

Both groups showed statistically significant positive effects (ADHD: β = 0.712, *p* = 0.002; TDC: β = 0.636, *p* = 0.002), suggesting that increased attentional engagement supports better emotional regulation. H3 is supported.

*H4*: Cognitive Load → Emotion Regulation

Negative effects were observed in both groups (ADHD: β = –0.547; TDC: β = –0.240, *p* < 0.05), affirming that greater cognitive burden negatively impacts emotional regulation. H4 is supported.

*H5*: Emotion Regulation → Behavioral Outcome

Significant negative associations were detected in both groups (ADHD: β = –0.358, *p* = 0.012; TDC: β = –0.992, *p* = 0.000), indicating that reduced emotional stability hinders behavioral improvement. H5 is supported.

*H6*: Learning Motivation → Emotion Regulation

Despite being statistically significant (*p* < 0.001), this path showed a negative coefficient, suggesting that overly elevated motivation might exert emotional strain, thereby impairing regulation. H6 is supported.

*H7a*: Cognitive Load × Learning Motivation → Emotion Regulation

Significant moderation effects emerged across both groups (*p* < 0.01). The ADHD group exhibited a positive interaction effect (β = 0.253), while the TDC group showed a negative effect (β = –0.077), suggesting group-specific moderation mechanisms. H7a is partially supported.

*H7b*: Cognitive Load × Attention Focus → Emotion Regulation

Significant interactive effects were detected in both groups (ADHD: β = –0.117, *p* = 0.000; TDC: β = –0.019, *p* = 0.002), confirming that attention and cognitive load jointly influence emotional regulation. H7b is supported.

In conclusion, the multi-group path analysis revealed that most hypothesized relationships were statistically significant in both groups, supporting a common foundational mechanism underlying the AR picture book intervention. Nonetheless, notable intergroup variations in effect direction and magnitude—particularly in AR engagement and interaction terms—highlight the need for population-specific adaptation. These results underscore the theoretical and practical importance of exploring psychological and behavioral heterogeneity through a multivariate lens, offering valuable guidance for optimizing cross-group educational strategies and designing personalized learning interventions for neurodiverse learners.

## 6 Discussion

### 6.1 Interpretation of key findings

This study suggests that augmented reality (AR) picture books can support attention regulation, learning motivation, and emotional control in children diagnosed with ADHD. The proposed structural path model reveals a sequential mechanism in which AR engagement stimulates learning motivation, which subsequently improves attentional focus and facilitates emotional regulation. These findings substantiate the theoretical assumption that motivation serves as a foundational precursor to emotional self-regulation in educational settings. However, given the relatively small sample size and the 2-week duration of the intervention, these conclusions should be interpreted as preliminary and may require further validation in larger, longitudinal studies.

### 6.2 Comparison with prior studies

The present findings are consistent with earlier research on AR-based educational interventions (Tosto et al., 2021; Doulou and Drigas, 2022), especially studies emphasizing the positive impact of multisensory input and interactive narrative structures. Unlike prior studies that concentrated mainly on reading performance or persistence in task completion, this research establishes a structured cognitive–affective–behavioral mechanism, empirically validated via Partial Least Squares Structural Equation Modeling (PLS-SEM). Furthermore, by introducing perceived cognitive load as a moderating factor, this study provides a novel analytical lens for understanding how task complexity and processing demands influence emotional regulation—particularly in neurodiverse populations.

Specifically, Tosto et al. (2021) found that AR narrative tools improved literacy and attentional engagement, which aligns with our conclusion that task-structured AR experiences effectively enhance learning motivation. Likewise, Doulou and Drigas (2022) emphasized the role of gamified AR environments in promoting metacognitive skills and emotional self-regulation—mechanisms that are also validated within our model. Lastly, our identification of cognitive load as a moderating variable echoes the work of Goharinejad et al. (2022), who highlight the role of sensory overload and individual differences in executive function in shaping the efficacy of extended reality (XR) interventions.

### 6.3 Reflections on mechanisms and group differences

One of the most striking findings was the divergence between the ADHD and typically developing (TDC) groups, particularly in the path from AR engagement to attentional focus. For TDC participants, AR use significantly enhanced attention, while for children with ADHD, the same interaction produced a negative path coefficient. This inverse relationship suggests that, without careful pacing and sensory modulation, AR experiences may overwhelm rather than support the attentional capacities of neurodiverse learners. Such asymmetry highlights the critical need for perceptual sensitivity, individualized pacing, and adaptive scaffolding in the design of AR educational tools.

### 6.4 Design implications for AR interventions

From a design standpoint, the findings offer valuable implications for developing AR-based educational interventions tailored to neurodiverse populations. First, the pacing of content delivery and the intensity of interactivity should be dynamically adjustable based on each learner’s cognitive threshold and attentional bandwidth. Second, feedback mechanisms must be carefully aligned with students’ self-regulation capacities—excessive stimuli may suppress rather than enhance emotional engagement. Third, embedding real-time emotion monitoring or cognitive load estimation features could facilitate adaptive interventions that better support learners with diverse needs. These design principles are essential for promoting the inclusive application of AR technologies in educational settings.

## 7 Conclusion

### 7.1 Theoretical contribution

Grounded in Self-Determination Theory (SDT) and emotion regulation theory, this study introduced AR-based digital picture books as a novel intervention strategy and developed a validated path model to examine psychological regulation in children with ADHD. The proposed model delineates a multi-stage process—AR engagement → learning motivation → attentional focus → emotion regulation → behavioral improvement—thereby establishing a structural theoretical framework for AR-supported educational interventions within special education contexts. A key theoretical contribution is the identification of learning motivation as a central mediating variable, a factor that has received limited systematic exploration in previous empirical studies.

Building on prior findings (e.g., Moradi Siah Afshadi et al., 2024), which emphasize the influence of motivation on executive function and emotional regulation among ADHD learners, the present study demonstrates that learning motivation functions as a cross-level bridge—exerting its impact on emotional regulation through attentional mediation. At the moderation level, the study introduces perceived cognitive load as a key contextual variable within AR-mediated learning environments. Empirical findings revealed that higher levels of perceived cognitive load negatively affect emotion regulation, particularly among children with ADHD—findings that align with Zhao and Zhang (2024).

Multi-group path modeling (PLS-MGA) further revealed that the moderating effects of cognitive load vary significantly between neurotypical and ADHD populations, thus advancing theoretical understanding of individualized learning thresholds in educational technology. Finally, multi-group comparison results revealed a divergence in the AR engagement → attention path: a strong positive effect among typically developing children contrasted with a negative effect among children with ADHD. This divergence underscores the importance of cognitively calibrated and perceptually adaptive AR design, especially when targeting neurodiverse learners. These findings extend the work of Tzang et al. (2022), who cautioned against presuming homogeneous emotional responses in gamified interventions for children with ADHD.

### 7.2 Practical implications

The 2-week AR picture book intervention led to significant improvements in attentional focus, learning motivation, and emotion regulation among children with ADHD, underscoring the practical utility of immersive and interactive technologies in special education settings. These findings offer a scalable framework for integrating digital technology into ADHD intervention strategies. Embedding AR content within narrative-driven and interactive frameworks not only increased learner engagement but also activated meaningful behavioral responses in children facing challenges with attention and emotional regulation.

The use of real-time feedback and multimodal inputs created a multisensory learning environment that supported both psychological involvement and behavioral participation. This intervention model demonstrates adaptability across multiple educational contexts, including special education classrooms, rehabilitation training programs, and home-based learning environments. Findings from the validated PLS-SEM model offer structured insights for educators and psychological support professionals.

For example, the study highlights the importance of monitoring the dynamic feedback loop between motivation and emotion regulation, ensuring that task difficulty is appropriately calibrated to each child’s cognitive and emotional thresholds to avoid overload. Similarly, AR content should be dynamically customized based on learners’ cognitive profiles to foster a “cognitive fit plus feedback enhancement” cycle. More importantly, the study identified group-specific differences in response to the same AR intervention mechanism. This finding confirms the necessity of moving beyond one-size-fits-all interventions, advocating instead for a “group differentiation → mechanism targeting → feedback optimization” framework guided by multi-group structural modeling.

### 7.3 Limitations

Despite offering valuable insights, this study has several limitations that warrant consideration in future research.

First, the sample was geographically and institutionally constrained, involving participants from a single region and two specific school types, with a relatively narrow age range and limited cultural diversity. These factors may limit the external validity and generalizability of the findings to broader populations. Second, the intervention spanned only 2 weeks, which limits the ability to evaluate sustained improvements in self-regulation and long-term behavioral transfer. Future studies should adopt longitudinal designs to assess the durability and scalability of such interventions.

Third, although teacher ratings were incorporated to improve data objectivity, the study primarily relied on self-reported measures. The absence of multimodal behavioral or physiological data—such as electroencephalography (EEG), eye-tracking, or naturalistic observation—limited the depth and precision of insight into participants’ emotional and attentional processes.

Fourth, it is important to acknowledge potential alternative explanations for the findings. The observed improvements in motivation and attention might be partially attributed to the “novelty effect” associated with the introduction of AR technology, rather than the educational content itself. Future longitudinal studies are needed to determine if these gains sustain once the novelty wears off. Additionally, while teacher ratings provide ecological validity, they may be subject to expectancy bias, as teachers were aware of the intervention status. Future research could benefit from including blinded assessors to mitigate this potential bias.

Lastly, the proposed model did not incorporate socio-emotional moderators such as social support, classroom climate, or self-efficacy—factors empirically linked to emotion regulation (Nguyen, 2023). Integrating these variables into future multi-layered structural models would offer a more comprehensive understanding of the mechanisms and contextual influences shaping intervention outcomes.

### 7.4 Future directions

Building upon the present findings, several promising directions for future research are proposed:

First, future AR interventions should be expanded into cross-platform, multimodal systems that integrate virtual reality (VR), haptic feedback, and other sensory modalities. Incorporating real-time behavioral tracking via online platforms could enhance ecological validity and support dynamically personalized interventions. Second, feedback mechanisms should be refined by introducing outer-loop regulatory processes, enabling the construction of closed-loop systems that dynamically align intervention content with individual psychological thresholds. This approach would facilitate the development of AI-supported personalized learning and therapeutic platforms.

However, transitioning from controlled experiments to real-world application requires addressing practical limitations. The cost of AR-capable devices and the need for ongoing technical support may limit accessibility in under-resourced educational settings. Furthermore, when discussing AI-supported personalization, educators face challenges related to infrastructure readiness and the need for specialized training to effectively integrate these advanced tools into daily instruction.

Third, the cross-cultural validity of the proposed model should be tested by broadening participant samples to include children from diverse sociocultural and educational backgrounds. Such comparative studies could uncover how cultural contexts shape the effectiveness and underlying mechanisms of AR-based interventions.

Finally, future efforts should promote a triadic collaboration framework that connects schools, families, and technological platforms. Given the multifactorial nature of ADHD-related challenges, interventions should actively involve both educators and caregivers. Embedding the model into classroom instruction and home guidance could foster continuity, enhance home–school synergy, and support the translational application of research findings in authentic educational settings.

## Data Availability

The original contributions presented in this study are included in this article/supplementary material, further inquiries can be directed to the corresponding author.

## References

[B1] Akçayır M. Akçayır G. (2017). Advantages and challenges associated with augmented reality for education: A systematic review. *Educ. Res. Rev.* 20 1–11. 10.1016/j.edurev.2016.11.002

[B2] American Psychiatric Association. (2013). *Diagnostic and statistical manual of mental disorders*, 5th Edn. Washington, DC: American Psychiatric Publishing.

[B3] Azuma R. T. (1997). A survey of augmented reality. *Presence* 6 355–385.

[B4] Bandura A. (1977). Self-efficacy: Toward a unifying theory of behavioral change. *Psychol. Rev.* 84 191–215. 10.1037/0033-295X.84.2.191 847061

[B5] Bandura A. (1997). *Self-efficacy: The exercise of control.* New York, NY: W. H. Freeman.

[B6] Barkley R. A. (2015). *Attention-deficit hyperactivity disorder: A handbook for diagnosis and treatment*, 4th Edn. New York, NY: Guilford Press.

[B7] Barroso A. González-López ÓR. Sanguino R. Buenadicha-Mateos M. (2018). Analysis and evaluation of the largest 500 family firms’ websites through PLS-SEM technique. *Sustainability* 10:557. 10.3390/su10020557

[B8] Billinghurst M. Clark A. Lee G. (2015). A survey of augmented reality. *Foundations Trends^®^ Human Comput. Interaction* 8 73–272. 10.1561/1100000049

[B9] Boyask R. Harrington C. Milne J. Smith B. (2023). “Reading enjoyment” is ready for school: Foregrounding affect and sociality in children’s reading for pleasure. *N. Zealand J. Educ. Stud.* 58 169–182. 10.1007/s40841-023-00265-z

[B10] Castles A. Rastle K. Nation K. (2018). Ending the reading wars: Reading acquisition from novice to expert. *Psychol. Sci. Public Interest* 19 5–51. 10.1177/1529100618772271 29890888

[B11] Champ R. E. Adamou M. Tolchard B. (2023). Seeking connection, autonomy, and emotional feedback: A self-determination theory of self-regulation in attention-deficit hyperactivity disorder. *Psychol. Rev.* 130 569–603. 10.1037/rev0000398 36548057

[B12] Cheah J. H. Thurasamy R. Memon M. A. Chuah F. Ting H. (2020). Multigroup analysis using SmartPLS: Step-by-step guidelines for business research. *Asian J. Bus. Res.* 10 1–19. 10.14707/ajbr.200087

[B13] Chen S. Y. Tseng W. T. Su Y. H. (2022). Investigating the role of support in students’ augmented reality-based learning: A multiple mediation model. *Front. Psychol.* 13:897405. 10.3389/fpsyg.2022.897405

[B14] Cheng K. H. Tsai C. C. (2014). Children and parents’ reading of an augmented reality picture book: Analyses of behavioral patterns and cognitive attainment. *Comput. Educ.* 72 302–312. 10.1016/j.compedu.2013.11.003

[B15] Chin W. W. Dibbern J. (2010). “An introduction to partial least squares,” in *Handbook of partial least squares*. Heidelberg: Springer, 1–32

[B16] China Education Online. (2024). *A survey report on the current situation and needs of Chinese children and adolescents in reading.* Available online at: https://www.eol.cn/news/yaowen/202312/t20231229_2552094.shtml (accessed March 15, 2025).

[B17] Danielson M. L. Visser S. N. Chronis-Tuscano A. DuPaul G. J. (2018). A national description of treatment among United States children and adolescents with attention-deficit/hyperactivity disorder. *J. Pediatrics* 192 240–246. 10.1016/j.jpeds.2017.08.040 29132817 PMC5732840

[B18] Doulou A. Drigas A. (2022). Electronic, VR & augmented reality games for intervention in ADHD. *Technium Soc. Sci. J.* 28 159–169. 10.47577/tssj.v28i1.5728

[B19] Dunleavy M. Dede C. (2014). “Augmented reality teaching and learning,” in *Handbook of research on educational communications and technology*, eds Spector J. M. (Berlin: Springer), 735–745. 10.1007/978-1-4614-3185-5_59

[B20] Durlak J. A. Weissberg R. P. Dymnicki A. B. Taylor R. D. Schellinger K. B. (2011). The impact of enhancing students’ social and emotional learning: A meta-analysis. *Child Dev.* 82 405–432. 10.1111/j.1467-8624.2010.01564.x 21291449

[B21] Ghalebandi S. G. Noorhidawati A. (2019). Electronic storybooks, print storybooks, and children’s engagement: An eye-tracking study. *J. Librariansh. Inf. Sci.* 51 779–791. 10.1177/0961000617742450

[B22] Goharinejad S. Ramezani M. Mousavi S. M. (2022). The usefulness of virtual, augmented, and mixed reality technologies for children with ADHD: A comprehensive overview. *BMC Psychiatry* 22:20. 10.1186/s12888-021-03632-1 34983446 PMC8728980

[B23] Gudinavičius A. Markelevičiūtė G. (2020). Using augmented reality in book publishing from a small language market perspective. *Publishing Res. Quart.* 36 43–54. 10.1007/s12109-020-09728-1

[B24] Harpin V. A. (2005). The effect of ADHD on the life of an individual, their family, and community from preschool to adult life. *Arch. Dis. Childh.* 90 i2–i7. 10.1136/adc.2004.059006 15665153 PMC1765272

[B25] Hautala J. Salmerón L. Tolvanen A. Loberg O. Leppänen P. (2022). Task-oriented reading efficiency: Interplay of general cognitive ability, task demands, strategies and reading fluency. *Read. Writ.* 35 1787–1813. 10.1007/s11145-021-10246-z

[B26] He S. Shuai L. Wang Z. Qiu M. Wilson A. Xia W. (2021). Online learning performances of children and adolescents with attention deficit hyperactivity disorder during the COVID-19 pandemic. *INQUIRY J. Health Care Organ. Provision Financing* 58:00469580211049065. 10.1177/00469580211049065 34647508 PMC8524690

[B27] Henseler J. Ringle C. M. Sinkovics R. R. (2009). “The use of partial least squares path modeling in international marketing,” in *New challenges to international marketing*. Emerald Group Publishing Limited, 277–319.

[B28] Hulme C. Snowling M. J. (2013). Learning to read: What we know and what we need to understand better. *Child Dev. Perspect.* 7 1–5. 10.1111/cdep.12005 26290678 PMC4538787

[B29] Keil M. Tan B. C. Y. Wei K. K. Saarinen T. Tuunainen V. Wassenaar A. (2000). A cross-cultural study on escalation of commitment behavior in software projects. *MIS Q.* 24 299–325.

[B30] Keshav N. U. Vogt-Lowell K. Vahabzadeh A. Sahin N. T. (2019). Virtual reality game: Significant correlation between student game performance and validated clinical measures of ADHD. *Children* 6:72. 10.3390/children6060072 31142022 PMC6617061

[B31] Kucuk S. Kapakin S. Goktas Y. (2016). Learning anatomy via mobile augmented reality: Effects on achievement and cognitive load. *Anatomical Sci. Educ.* 9 411–421. 10.1002/ase.1603 26950521

[B32] Marôco J. (2021). What makes a good reader? Worldwide insights from PIRLS 2016. *Read. Writ.* 34 231–272. 10.1007/s11145-020-10068-0

[B33] Mayer R. E. (2005). *The Cambridge handbook of multimedia learning.* Cambridge: Cambridge University Press.

[B34] Mayes S. D. Calhoun S. L. Crowell E. W. (2000). Learning disabilities and ADHD: Overlapping spectrum disorders. *J. Learn. Disabil.* 33 417–424. 10.1177/002221940003300502 15495544

[B35] McMahon D. Cihak D. F. Wright R. E. Bell S. M. (2016). Augmented reality as a navigation tool to employment opportunities for postsecondary education students with intellectual disabilities and autism. *J. Res. Technol. Educ.* 48 38–56. 10.1080/15391523.2015.1103148

[B36] Mitsea E. Drigas A. Skianis C. (2023). VR gaming for meta-skills training in special education: The role of metacognition, motivations, and emotional intelligence. *Educ. Sci.* 13:639. 10.3390/educsci13070639

[B37] Moradi Siah Afshadi M. Amiri S. Talebi H. (2024). Examining the structural equation modeling between intrinsic-motivation, emotion regulation and ADHD: The mediating role of problem-solving, time-management. *Curr. Psychol*. 10.1007/s12144-023-04289-7 Online ahead of print. 36820196 PMC9931447

[B38] Nguyen T. A. (2023). *Child emotion-regulation and parent psychological flexibility following an integrated ACT-PMT intervention for ADHD.* Houston, TX: University of Houston Clear Lake Institutional Repository.

[B39] Obradović J. Portilla X. A. Ballard P. J. (2012). Biological sensitivity to context in Latino children: The moderating role of child care quality. *Child Dev.* 83 1742–1756. 10.1111/j.1467-8624.2012.01811.x 22966919 PMC3443076

[B40] Park H. Kim S. Han J. (2023). Attention-focused task design in immersive learning environments: Effects on ADHD learner performance. *Br. J. Educ. Technol.* 54 327–342. 10.1111/bjet.13295

[B41] Piotrowski J. T. Krcmar M. (2017). Reading with hotspots: Young children’s responses to touchscreen stories. *Comput. Hum. Behav.* 70 328–334. 10.1016/j.chb.2017.01.002

[B42] Psyrra G. Mangina E. Treacy R. (2023). “Case study of AR digital literacy intervention for students diagnosed with ADHD,” *In Mixed Reality for Education*, eds Cai Y. Mangina E. Goei S. L. (Berlin: Springer), 291–313. 10.1007/978-981-99-1494-2_14

[B43] Radu I. (2014). Augmented reality in education: A meta-review and cross-media analysis. *Personal Ubiquitous Comput.* 18 1533–1543. 10.1007/s00779-013-0747-y

[B44] Ronimus M. Tolvanen A. Hautala J. (2022). The roles of motivation and engagement in computer-based assessment of children’s reading comprehension. *Learn. Individ. Dif.* 98:102197. 10.1016/j.lindif.2022.102197

[B45] Rueda M. R. Posner M. I. Rothbart M. K. (2005). The development of executive attention: Contributions to the emergence of self-regulation. *Dev. Neuropsychol.* 28 573–594. 10.1207/s15326942dn2802_2 16144428

[B46] Ryan R. M. Deci E. L. (2000). Self-determination theory and the facilitation of intrinsic motivation, social development, and well-being. *Am. Psychol.* 55 68–78. 10.1037/0003-066X.55.1.68 11392867

[B47] Ryan R. M. Deci E. L. (2017). *Self-determination theory: Basic psychological needs in motivation, development, and wellness.* New York, NY: The Guilford Press, 10.1521/978.14625/28806

[B48] Sankaranarayanan A. Bhuvaneswari S. Lakshmi P. (2022). Virtual reality intervention for attention deficit hyperactivity disorder: A brief review. *Children* 9:567. 10.3390/children9040567 35455611 PMC9033145

[B49] Sanku D. George R. Thomas R. (2023). Augmented reality interventions for ADHD: A systematic review. *J. Educ. Comput. Res.* 61 1435–1464. 10.1177/07356331231163520

[B50] Simon V. Czobor P. Bálint S. Mészáros Á Bitter I. (2009). Prevalence and correlates of adult attention-deficit hyperactivity disorder: Meta-analysis. *Br. J. Psychiatry* 194 204–211. 10.1192/bjp.bp.107.048827 19252145

[B51] Şimşek M. Direkçi M. (2023). The effect of AR-based storybooks on early literacy skills and motivation in preschool children. *Educ. Inf. Technol.* 28 7655–7673. 10.1007/s10639-023-11685-w 37361765 PMC10026236

[B52] Sulaiman T. Shaid M. (2023). The effectiveness of immersive media-based learning on emotional regulation of ADHD children. *Int. J. Inclusive Educ.* 10.1080/13603116.2023.2203472 Advance online publication.

[B53] Sweller J. Ayres P. Kalyuga S. (2011). *Cognitive load theory.* Berlin: Springer, 10.1007/978-1-4419-8126-4

[B54] Teglasi H. Schussler L. Gifford K. Annotti L. A. Sanders C. Liu H. (2015). Child behavior questionnaire–short form for teachers: Informant correspondences and divergences. *Assessment* 22 730–748. 10.1177/1073191114562828 25573857

[B55] Thapar A. Cooper M. Jefferies R. Stergiakouli E. (2012). What causes attention deficit hyperactivity disorder? *Arch. Dis. Childh.* 97 260–265. 10.1136/archdischild-2011-300482 21903599 PMC3927422

[B56] Tosto C. Hasegawa T. Mangina E. Chifari A. Treacy R. Merlo G. (2021). Exploring the effect of an augmented reality literacy programme for reading and spelling difficulties for children diagnosed with ADHD. *Virtual Reality* 25 879–894. 10.1007/s10055-020-00485-z

[B57] Tzang R. F. Chang C. H. Chang Y. C. (2022). Structural equation modeling (SEM): Gaming disorder leading untreated attention-deficit/hyperactivity disorder to disruptive mood dysregulation. *Int. J. Environ. Res. Public Health* 19:6648. 10.3390/ijerph19116648 35682233 PMC9179962

[B58] Tzima S. Styliaras G. Bassoukos A. (2019). Interactive storytelling in education: The impact of augmented reality on storytelling. *Educ. Information Technol.* 24 1295–1316. 10.1007/s10639-018-9831-z

[B59] Welch B. L. (1947). The generalization of ‘Student’s’ problem when several different population variances are involved. *Biometrika* 34 28–35. 20287819 10.1093/biomet/34.1-2.28

[B60] Whipp A. M. Vuoksimaa E. Bolhuis K. de Zeeuw E. L. Korhonen T. Mauri M. (2021). Teacher-rated aggression and co-occurring behaviors and emotional problems among schoolchildren in four population-based European cohorts. *PLoS One* 16:e0238667. 10.1371/journal.pone.0238667 33914742 PMC8084195

[B61] Wu H. K. Lee S. W. Y. Chang H. Y. Liang J. C. (2013). Current status, opportunities and challenges of augmented reality in education. *Comput. Educ.* 62 41–49. 10.1016/j.compedu.2012.10.024

[B62] Yang S. Lee J. Park Y. (2025). Enhancing children’s comprehension through interactive AR storytelling: Task-based design approaches. *Interactive Learn. Environ.* 10.1080/10494820.2025.2033888 Advance online publication.

[B63] Yeh Y. C. Lin C. H. Chen Z. H. (2022). Improving children’s reading engagement and motivation with augmented reality storybooks: Evidence from a longitudinal study. *Comput. Educ.* 180:104429. 10.1016/j.compedu.2022.104429

[B64] Yilmaz R. M. Kucuk S. Goktas Y. (2021). Effects of AR storybooks on reading attitude and storytelling performance in early childhood. *Educ. Inf. Technol.* 26 745–765. 10.1007/s10639-020-10287-6

[B65] Yoon S. Wang H. (2020). Using augmented reality and virtual reality to improve engagement and comprehension in early literacy classrooms. *Comput. Educ.* 147:103784. 10.1016/j.compedu.2019.103784

[B66] Zhao D. Zhang J. (2024). Effects of memory training on attention deficit, adaptive and non-adaptive cognitive emotion regulation of Chinese children with ADHD. *BMC Psychol.* 12:1539. 10.1186/s40359-024-01539-6 38317179 PMC10845547

[B67] Zheng H. Dong Y. Sun Y. K. Yang J. Yuan C. (2021). Effectiveness of metacognitive regulation intervention on attention-deficit–hyperactivity disorder students’ scientific ability and motivation. *Front. Psychol.* 12:747961. 10.3389/fpsyg.2021.747961 35002845 PMC8732764

[B68] Zheng R. Z. (2022). *Influence of multimedia and cognitive strategies in deep and surface verbal processing: A verbal-linguistic intelligence perspective.* Palmdale: IGI Global.

[B69] Zhou N. Yadav A. (2017). Effects of multimedia learning anxiety on children’s engagement and learning outcomes in augmented reality. *J. Educ. Comput. Res.*, 55 76–100.

[B70] Zima B. T. Bussing R. Belin T. R. Forness S. R. (2000). Use of mental health services by children in foster care in Los Angeles County: A study of racial/ethnic disparity. *J. Am. Acad. Child Adolesc. Psychiatry* 39 1143–1151. 10.1097/00004583-200010000-00006 11026173

